# Ochratoxins in Feed, a Risk for Animal and Human Health: Control Strategies

**DOI:** 10.3390/toxins2051065

**Published:** 2010-05-13

**Authors:** Muzaffer Denli, Jose F. Perez

**Affiliations:** 1 Department of Animal Science, Faculty of Agriculture, Dicle University, 21280, Diyarbakir, Turkey; Email: muzaffer.denli@gmail.com; 2 Departament de Ciència Animal i dels Aliments, Facultat de Veterinària, Universitat Autònoma de Barcelona, 08193 Bellaterra, Barcelona, Spain

**Keywords:** ochratoxins, toxicity, human, animals, control strategies

## Abstract

Ochratoxin A (OTA) has been shown to be a potent nephrotoxic, hepatotoxic, and teratogenic compound. In farm animals, the intake of feed contaminated with OTA affects animal health and productivity, and may result in the presence of OTA in the animal products. Strategies for the control of OTA in food products require early identification and elimination of contaminated commodities from the food chain. However, current analytical protocols may fail to identify contaminated products, especially in animal feed. The present paper discusses the impact of OTA on human and animal health, with special emphasis on the potential risks of OTA residue in animal products, and control strategies applied in the feed industry.

## 1. Introduction

Ochratoxin A (OTA) is one of the several fungal mycotoxins that have aroused significant public concern worldwide. The disease caused by OTA exposure is known as *ochratoxicosis*, and the primary target is the kidney. Epidemiological studies show that OTA may be involved in the pathogenesis of different forms of human nephropathies, including kidney cancer [1,2,3]. Tumor incidence data from long-term animal studies also provides reasons for concern about the effect of OTA exposure on the human population. Thus, OTA was classified as a possible carcinogen (Group 2B) to humans by The International Agency for Research on Cancer (IARC) [4]. The mechanism of action of OTA is unclear. Recent reports suggest that oxidative pathway and genotoxicity are the key points for both nephrotoxicity and carcinogenity [3]. Degenerative changes of the epithelial cells of the kidneys and the liver could be explained by the route of elimination of OTA via the kidneys and partly via the liver [5].

OTA may be encountered in a host of common foodstuff and beverages. The highest reported occurrence of OTA was found in cereal grains, and to a lower extent in other foodstuff of plant origin (*i.e.*, wine, coffee, beer, spices and chocolate). Moreover, considering that mycotoxins can be transferred through the food chain, OTA can also be found in tissues and products of animal origin, pork and poultry, and dairy products, among others [6,7]. 

This article provides a review of the natural occurrence of OTA in animal feed, and an update of the human exposure to OTA contaminated food of animal origin, mitigation practices in the feed industry, and regulatory measures being taken in Europe.

## 2. Natural Occurrence of OTA in Animal Feed

OTA is a secondary toxic metabolite produced mainly by some strains of *Aspergillus ochraceus* and *Penicillium verrucosum *species. These species can grow in different climates. *Aspergillus* are found in tropical regions, whereas *Penicillia* are common in temperate regions; and can grow when the temperature is as low as 5 ºC [8]. In general, OTA formation occurs mainly after harvesting on insufficiently dried cereal and cereal products. Factors influencing OTA production include environmental conditions, such as temperature and water activity, but also the type and integrity of the seeds. While *A. ochraceus *grows better in oilseeds (peanuts and soybeans) than in grain crops, such as wheat and corn, *P. verrucosum* may grow better in wheat and corn [9]. A wide variety of nutritional based biotic factors may affect the production of OTA biosynthesis. While, different carbon sources, including glucose, sucrose, galactose or xylose, appear to repress OTA production in *A. ochraceus*; other compounds, such as lactose, and organic nitrogen, such as urea and amino acids, induces its production [10].

OTA has been found in cereal grains (maize, barley, wheat, oats, rye), hay and mixed feed [11,12]. OTA amount in animal feed varies from country to country. The highest amounts have been reported in Northern Europe and North America [8]. Specifically, the highest frequencies were described in Denmark (57.6%), Canada (56.3%) and Yugoslavia (25.7%), showing isolated samples with values of OTA contamination above 5,000 µg/kg. However, until recent reports, no data were available from many countries, particularly from Asia and South America [13]. In a study carried out in Spain with a large number of ingredients sampled from the same feed mill, Espada [14] found the highest levels of OTA (above the levels recommended by the EU legislation (2006/576/CE; <50 µg/kg)) in a reduced number of samples (1%) of maize (up to 225 µg/kg) and barley (up to 90 µg/kg). In Brazil, Rosa *et al* [15] described the OTA occurrence in corn, brewers grain and finished swine feed samples collected from different factories. Corn samples (44%) were contaminated with 42-224 µg/kg of OTA. The animal feed (31%) and samples of brewers grain (13%) were contaminated with 36-120 µg/kg and 28-139 µg/kg of OTA, respectively. In Argentina, Dalcero *et al* [16] detected also OTA in 38% of the poultry feed samples tested with levels ranging from 25 to 30 µg/kg; in 25% of rabbit feed samples, with levels ranging from 18.5 to 25 µg/kg; and in 13% of the pig feed samples with similar levels of toxins.

## 3. Effects of OTA on Animal Production and Health

OTA-contaminated feed has its major economic impact on monogastric animals and the poultry and pig industry. Ruminant animals are more resistant than monogastrics to OTA toxicity. In general, exposure to OTA contaminated feed reduces animal growth rates and affects animal production. Pigs are generally considered as the animal species most sensitive to the nephrotoxicity of OTA [12]. Nephropathy, but without renal failure, was observed in female pigs fed on diets containing 1 mg OTA/kg feed, but not on diets containing 0.2 mg OTA/kg feed for two years [12]. Degenerative changes affecting epithelial cells in some proximal tubules were observed in pigs given a diet containing OTA at 0.8 mg/kg for six months, as well as proliferative changes in the interstitium, which predominated after one year [17]. The phagocytic activity and the production of IL2 were decreased when pigs consumed feed contaminated by OTA (2.5 mg/kg) [18]. In another pig study, ingestion of feed containing 25 μg/kg decreased feed efficiency, daily gain weight and final body weight [19].

The poultry industry is also affected by OTA contamination. Turkeys, chickens and ducklings are susceptible to this toxin. Typical signs of poultry ochratoxicosis are reduction in weight gain, poor feed conversion, reduced egg production, poor egg shell quality and nephrotoxicity. OTA fed at various doses (1-5 mg/kg), to animals of various ages, altered their serum biochemistry, including decreases in cholesterol, total protein, albumin, globulin, potassium, and triglyceride levels, and increases in uric acid and creatine levels and in the activities of serum alkaline phosphatase (ALP) and gamma glutamine transpeptidase (GGT) [20,21,22,23]. The effects depend on the level of the toxin and time exposure. However, numerous studies showed that even exposure to low levels of OTA (0.5 mg/kg feed) altered performance, including decreased feed consumption and growth rate and poor feed conversion efficiency [24,25]. Reduction in egg production and egg weight were recorded in laying hens when animals were fed a diet contaminated with OTA at 2 and 3 mg/kg levels [26,27]. We also reported that laying hens fed on a diet contaminated with 2 mg OTA/kg significantly reduced daily feed consumption, egg mass production, and serum triglyceride concentrations, and increased the relative liver weight as compared with a control diet [20]. Weight losses, diarrhea, excessive urine excretion and renal lesions have been noted in chickens fed a diet contaminated with 2 mg OTA/kg [28]. Exposure to OTA-contaminated feed (2.5 mg OTA/kg) also decreased the concentration of α-tocopherol in the chicken liver [29] and impaired chick immune function even at concentrations as low as 0.25 mg/kg of OTA [25].

## 4. OTA Presence in Animal Products

The presence of OTA in animal based foodstuff may occur as a result of both direct fungi contamination of the animal products or indirectly via contaminated feed, with the latter being the main problem. Following oral consumption of OTA-contaminated feed, rapid absorption in the blood via the gastrointestinal tract is generally followed by relatively slow elimination in urine and feces [30,31]. Vettorazzi *et al.* [32] also reported that, a single oral dose of OTA (500 μg/kg), caused the maximum plasma concentration within 2 h, accounting for ~30% of the OTA intake. Plasma half-life of OTA in males was 10 days. Versantvoort *et al.* [33] described bioaccesibility values of OTA from food of 100% in an *in vitro* digestion model. Efficient enterohepatic recycling of OTA and reabsorption by the renal tubules also contributes to the persistence of OTA in plasma [34,35].

Based on its polar composition, OTA is widely distributed throughout the body fluids, in organs and tissues of rats, pigs, rabbits and chickens [36,38]. Ferrufino-Guardia *et al.* [38] reported an effective transfer of OTA from blood to milk in lactating rabbits fed naturally-contaminated diet (mean milk/plasma concentration ratio of 0.015). At slaughter, the highest concentrations of OTA accumulated in the body of the rabbits were found in kidney followed by liver, mammary gland and muscle. OTA residue in pig meat was found to be up to 1 µg/kg when the growing pigs were fed a diet contaminated with OTA at 25 µg/kg [39]. In contrast, OTA residue does not generally accumulate to a significant extent in ruminants because OTA is rapidly detoxified by rumen protozoa and bacterial enzymes into less toxic metabolites [40]. Degradation of OTA in the rumen to less toxic OTα is considered a key point in the lower potential risk of OTA in ruminants [41]. However, a constant proportion (6−8%) of the unaltered OTA dose has been demonstrated to be excreted in the urine of sheep fed a diet contaminated with various doses (0, 9.5, 19.0, and 28.5 μg/kg body weight) of OTA [42].

In the pig industry, OTA is considered a main cause of porcine nephropathy [43]. In Denmark, the level of OTA in pork has been indirectly controlled by visual examination of kidneys from slaughtered pigs for macroscopic changes [44]. If there are changes, the kidneys will be analyzed. A condemnation of the entire carcass when OTA reaches 25 µg/kg in pig kidney is considered to ensure that the amounts of OTA in meat will not exceed 10 µg/kg. This is based on the estimation that the OTA content in pig meat is approximately 40% of the level in pig kidney [45]. Pig liver and pig kidney are rejected when the amount of OTA ranges between 10 to 25 μg/kg in kidney. In 1998 (after a very wet period during harvesting in 1987), 13.5% of the kidneys having OTA content above 25 μg/kg have induced whole carcass condemnation. In 1995, less than 1% of these organs contained more than 25 μg/kg. Year-to-year variations may reflect differences in the harvesting and climate conditions between years, but also changes of the storage conditions of the foodstuff. In a recent study in Serbia, Milicevic *et al.* [46] presented data on the occurrence of OTA contamination in liver and kidney samples from slaughtered pigs (N = 90). Of the 90 pigs, 26.6% of the liver samples contained OTA in the range of 0.22-14.5 µg/kg (mean 0.63 µg/kg), and 33% of the kidney samples in the range of 0.17-52.5 µg/kg (mean, 1.26 µg/kg). OTA has also been detected in meat and offal from pigs in France and Italy [47,48]. Jimenez *et al.* [49] evaluated the occurrence of OTA in 38 pig-derived pâtés. Three of them contained OTA above the detection limit of 0.56 µg/kg. The highest concentration (1.77 µg/kg) was found in a homemade pâté.

For milk, little information is available on the rate of transfer of this toxin into milk for dairy cows. In dairy sheep, the carryover is less than 1% [50]. In a survey conducted in the northwest of France in 2003, Boudra *et al.* [51] evaluated the presence of aflatoxin M_1_ (AFM_1_) and OTA in raw bulk milk (N = 132) from farms based on corn silage and cereal grains grown within the own farm, which represent the maximum risk of mycotoxin contamination. OTA was detected in three milk samples at low levels, 5-8 ng/L, at which they do not appear to pose any particular risk to milk consumers. The concentration of OTA in human or other non-ruminant mammal’s milk may be higher than ruminants [40]. 

Residues of OTA have been also detected in the muscle of hens and chickens, and in eggs [6]. In an experiment in which we evaluated the toxic effects of OTA (OTA, 2 mg/kg of feed) in laying hen diets, OTA exposure promoted an increase in the content of OTA in the liver (15.1 µg/kg) as compared to control animals [20]. However, OTA residue was not detected above our detection limit (0.05 µg/kg) in any of the analyzed eggs. Moreover, Tangni *et al.* [52] examined the presence of OTA in some home-produced eggs in Belgium, and observed that intake of OTA via the consumption of home produced eggs seems not to be a matter of concern. OTA is hardly detected in analyzed eggs, unless the animals are fed on high OTA contaminated feed. Niemiec *et al. * [53] observed a range from 0.7 to 1.3 μg OTA/kg of eggs when animals were fed a diet contaminated with OTA at 10.0 mg/kg. In contrast, no OTA was detected in eggs of Japanese quails given 1 mg OTA/kg BW [6].

## 5. Human Exposure to OTA Contaminated Food of Animal Origin

OTA occurrence in human food commodities of vegetal and animal origin has been recognized as a potentially global human health hazard. Several detailed risk assessments have linked kidney damage incidence to estimated OTA consumption in the diet [54]. OTA is associated with the Balkan Endemic Nephropathy and was also linked to human renal disease [55,57]. Moreover, it has been described that OTA is genotoxic and associations have been found between OTA exposure and tumor incidences in long-term animal bioassays [3,58]. A general maximum OTA limit of 5 µg/kg in cereals and 3 µg/kg in cereal products was proposed by the World Health Organization [8]. 

OTA has been detected in human blood and human milk samples [59,60]. The increase of OTA in several human fluids in the various populations of endemic regions may describe the human exposure to OTA contaminated food [3,57,61]. Thuvander *et al. *[62] determined blood levels of OTA in 406 Scandinavian blood donors. The authors described the strongest correlations (correlation coefficient r > 0.4; P < 0.001) for women in relation to the consumption of beer or medium brown bread. Consumption of several foodstuff, including cereal products, wine, beer and pork, were to a minor degree related to high plasma levels of OTA. 

In breast milk, Skaug *et al.* [63] found 17 (21%) out of 80 human milk samples containing OTA in the range 10-182 ng/L. However, the highest values were observed in a survey with samples from 75 mothers in Turkey [64]. OTA was found in all samples tested in the range of 0.620-13.11 µg/L. Galvano *et al.* [65] also detected OTA in 61 (74%) of 82 milk samples collected in Italian hospitals (ranging from < 5 ng/L to 405 ng/L; mean level: 30.43 ng/L). OTA levels were significantly higher (p < 0.05) in the milk of habitual consumers of bread, bakery products and cured pork meat. These results confirm the occurrence of OTA in human milk and its likely association with maternal dietary habits. The strongest associations were observed with foodstuff sources of vegetable origin and, to a lesser extent, with food of animal origin. The findings also support the possibility of issuing dietary recommendations to women during pregnancy and lactation, aimed at reducing the OTA contamination of human milk.

## 6. Regulation of OTA in Food

Complete elimination of any natural toxicant from foodstuff is an unattainable objective [66]. Therefore, many organizations and countries have established regulations or guidelines for tolerable levels of OTA in food and animal feed. The U.S. Food and Drug Administration (FDA), Food and Agricultural Organization of the United Nations (FAO), European Union (EU), the Institute of Public Health of Japan and many other local agencies have set a regulation and guidelines for the maximum tolerable limits of different mycotoxins. The European Commission [67] (Table 1) has established a regulation list for the maximum tolerable limits of OTA in foodstuff commodities, such as cereals (5 µg/kg), cereal products (3 µg/kg), wine and dried fruits (10 µg/kg) and foodstuff for baby and children below three years of age (0.5 µg/kg). Convinced by several sets of toxicological data from animal studies, the commission has also recommended that OTA levels be reduced to below 5 ng/kg of body weight per day [68]. 

**Table 1:** European Union Maximum level of ochratoxin permitted in foodstuff.

Commodities	Maximum level (µg/kg)
Raw cereals	5.0
Cereal products	3.0
Infant based food	0.5
Dried vine fruit	10.0
Roasted coffee beans	5.0
Soluble coffee	10.0
Wine and grape juice	2.0

The Commission of the European Communities [69] recommended guidelines for the maximum tolerable limits of different mycotoxins in feed, cereal and cereal products for animal feeding (Table 2).

**Table 2:** The Commission of the European Communities Recommendation (2006/576) guidance values for OTA in feedstuffs.

Feed Commodities	Maximum level (µg/kg)
Cereals and cereal products	250
Completary and complete feedstuffs for pigs	50
Completary and complete feedstuffs for poultry	100

## 7. Mitigating the Effects of OTA in the Animal Industry

Several different strategies have been employed to reduce the risk of OTA entering the animal feed industry or its transfer into the food chain. The main strategies address control of the growth of OTA producing fungi during the harvesting and storage period. For this purpose, some acids (*i.e.*, Propionate) are usually included in the feed to avoid growth of moulds and mycotoxin production. However, mycotoxin may contaminate the ingredients before arriving at the feed mills, due to the difficulties in controlling climate and environmental conditions. Rigorous risk management protocols in the feed mills to avoid the incorporation of contaminated grain into the animal feed, elimination of OTA from feedstuffs by using chemical and physical methods, or the administration of OTA contaminated diets to animal species that are less susceptible to OTA toxicity, are alternative strategies which have been considered by the feed industry [1].

The formal establishment of risk management protocols to identify mycotoxin contaminated ingredients is considered mandatory to obtain safe feed. However, mycotoxin analyses by the ELISA technique are not very sensitive. As an alternative, Near Infrared Spectroscopy (NIR) is likely to allow improved control strategies in the feed mills industry. As a precautionary step, it is important to ensure quality control of each batch to confirm the absence of matted products and/or grains corroded, broken or damaged by insects. 

Many physical methods, including high temperature or drying, have been carried out to detoxify OTA. However, OTA is a mycotoxin that is difficult to destroy once released in the feed. Temperatures up to 250 ºC for several minutes are required to destroy the OTA compounds in foodstuff [70]. Should OTA represent a risk in the feed, various treatment methods have been tested for eliminating or reducing its harmful effects on animals, including the use of specific adsorbents to block mycotoxin in the digestive content or microorganisms capable of biotransforming mycotoxins into nontoxic metabolites [20,71] and the use of antioxidant compounds [72]. 

The use of adsorbents, which bind OTA efficiently in the gastrointestinal tract, seems a promising and economical approach to reduce the negative effects of OTA in the animal industry. To this end, various adsorbents have been tested in both *in vitro* and *in vivo* models. Hydrated sodium calcium aluminosilicate [73,74], activated charcoal, bentonite and cholestyramine [75], and esterified glucomannan [76] have been used in animal feed to diminish the adverse effects of OTA. However, many of these agents have failed to prevent ochratoxicosis in animals. *In vivo* studies have demonstrated that aluminosilicates and many proposed adsorbents are capable of absorbing aflatoxins, but do not prevent the toxicity of dietary OTA [73,77]. Some adsorbents may be very effective in preventing mycotoxicosis induced by some toxins but their efficacy against others may be limited. Until now, no single adsorbent has been shown to be effective against most types of mycotoxins [78]. It is known that each mycotoxin has a different physical and chemical structure. Thus, adsorbents for a specific toxin should present specific binding properties, such as total charge and charge distribution, pore size and accessible surface area. Moreover, adsorbents showing high binding capacities *in vitro* may not exhibit the same effects in *in vivo* conditions [79].

Some other substances, such as antioxidants, have also been evaluated to decrease OTA toxicity in several species. Abdel-Wahhap *et al.* [80] and Özçelik *et al.* [81] found that melatonin exhibits a preventive effect against OTA-induced oxidative stress and structural damage in the kidney through its role in the scavenging of free radicals and/or the prevention of lipid peroxidation. Grosse *et al.* [82] also demonstrated that the incorporation of alpha-tocopherol in the diet decreased by 58% the total DNA adduct provoked in kidney by a single administration of OTA in mouse and rat kidney. In other studies, addition of a plant extract (artichoke extract) or sesame seed to laying hen diets showed protection against the suppressive effect of OTA on egg production and the toxic effect of OTA on various internal organs [83]. 

## 8. Conclusion

OTA contamination of food has aroused significant public concern worldwide. The main reasons for this concern arise from the fact that OTA is considered a nephrotoxic and carcinogenic agent, being at the origin of many kidney diseases. OTA is mainly found in cereal and oilseed grains, and to a minor extent in animal products. However, several strategies are being carried out in the animal feeding industry to prevent or reduce the contamination of the toxin in food and feed commodities. The most applicable methods until now are the establishment of risk management protocols to identify and prevent the incorporation of mycotoxin-contaminated ingredients into the animal feed. Current and coming alternatives include the use of binding agents designed to block the absorption of OTA from the gastrointestinal tract, or microorganisms and enzymes capable of biotransforming mycotoxins into nontoxic metabolites. 
